# Case report: Unpredictable nature of tubal cancer

**DOI:** 10.1016/j.amsu.2020.01.002

**Published:** 2020-01-16

**Authors:** Sigit Purbadi, Victor Prana Andika Santawi, Hartono Tjahjadi, Sahat Matondang, Laila Nuranna

**Affiliations:** a Department of Obstetrics and Gynaecology, Faculty of Medicine, Universitas Indonesia, Central Jakarta, Indonesia; b Department of Anatomical Pathology, Faculty of Medicine, Universitas Indonesia, Central Jakarta, Indonesia; c Department of Radiology, Faculty of Medicine, Universitas Indonesia, Central Jakarta, Indonesia

**Keywords:** Fallopian tube cancer, Endometrial cancer, Diagnostic, Case report

## Abstract

**Introduction:**

Primary tubal cancer is very rare, most are diagnosed intra and post operatively. Histopathology is vital in determining the cancer origin. Here we present a case of fallopian tube cancer with clinical presentation mimicking endometrial origin.

**Case description:**

A 74-year old patient came with complaints of intermittent post-menopausal bleeding and pelvic pain. The patient had several investigations using Ultrasonography, Hysteroscopy-guided biopsy, and Magnetic Resonance Imaging. Pre-operative diagnosis was endometrial cancer based on histopathology of endometrial biopsy during hysteroscopy. Explorative laparotomy, total abdominal hysterectomy, bilateral salphingo-oophorectomy, pelvic and para-aortic lymph node dissection were then performed, and the tumor samples were sent to the histopathology laboratory. It was found that the post-operative diagnosis was in fact primary fallopian tube cancer stage IIB.

**Conclusion:**

For patients with gynecological malignancies, rare cases such as fallopian tube cancer should never be overlooked as a differential diagnosis.

## Highlights

• 74 year old female, originally diagnosed as endometrial cancer.
• USG, Hysteroscopy biopsy, and MRI suggested endometrial cancer.
• The endometrial tumor is spillage of fallopian tumor.
• Final diagnosis was found to be Primary Fallopian Tube Cancer.

## 1 Introduction

Primary fallopian tube carcinoma (FTC) is a very rare gynecological malignancy. Despite being rare, most high-grade serous carcinomas arise from the fallopian [1]. Classic symptoms of FTC include vaginal bleeding or discharge and lower abdominal pain. Vaginal bleeding is the most commonly reported symptom of FTC and is present in approximately 50% of the patients. Other clinical symptoms suggestive of FTC include pelvic pain, watery vaginal discharge, and pelvic mass [2]. However, the pathognomonic Latzko's triad only present in 15% of the patients [3]. These non-specific symptoms often lead to misdiagnosis of FTC as endometrial cancer based on similar presentation and its higher epidemiological incidence. Most of the reported primary FTC cases were diagnosed intraoperatively or based on histopathological findings.

Here we report a rare case of primary fallopian tube carcinoma supported with intraoperative findings and histopathological examination. We hope to highlight the clinical presentation of FTC that is commonly underdiagnosed.

## 2 Case description

This Evidence Based Case-Report is made in line with the SCARE criteria [4].

A 74-year-old female came with post-menopausal bleeding lasting for a month. The bleeding occurred intermittently, approximately 1–2 pads each day. The patient also complained of intermittent pelvic tenderness which was more severe on the right side. Patient had 2 living children via spontaneous vaginal delivery with no abortion history, she had menopause 22 years ago. Past personal and familial medical history were unremarkable. Normal findings were found upon physical and gynecological examinations.

**Fig. 1:**
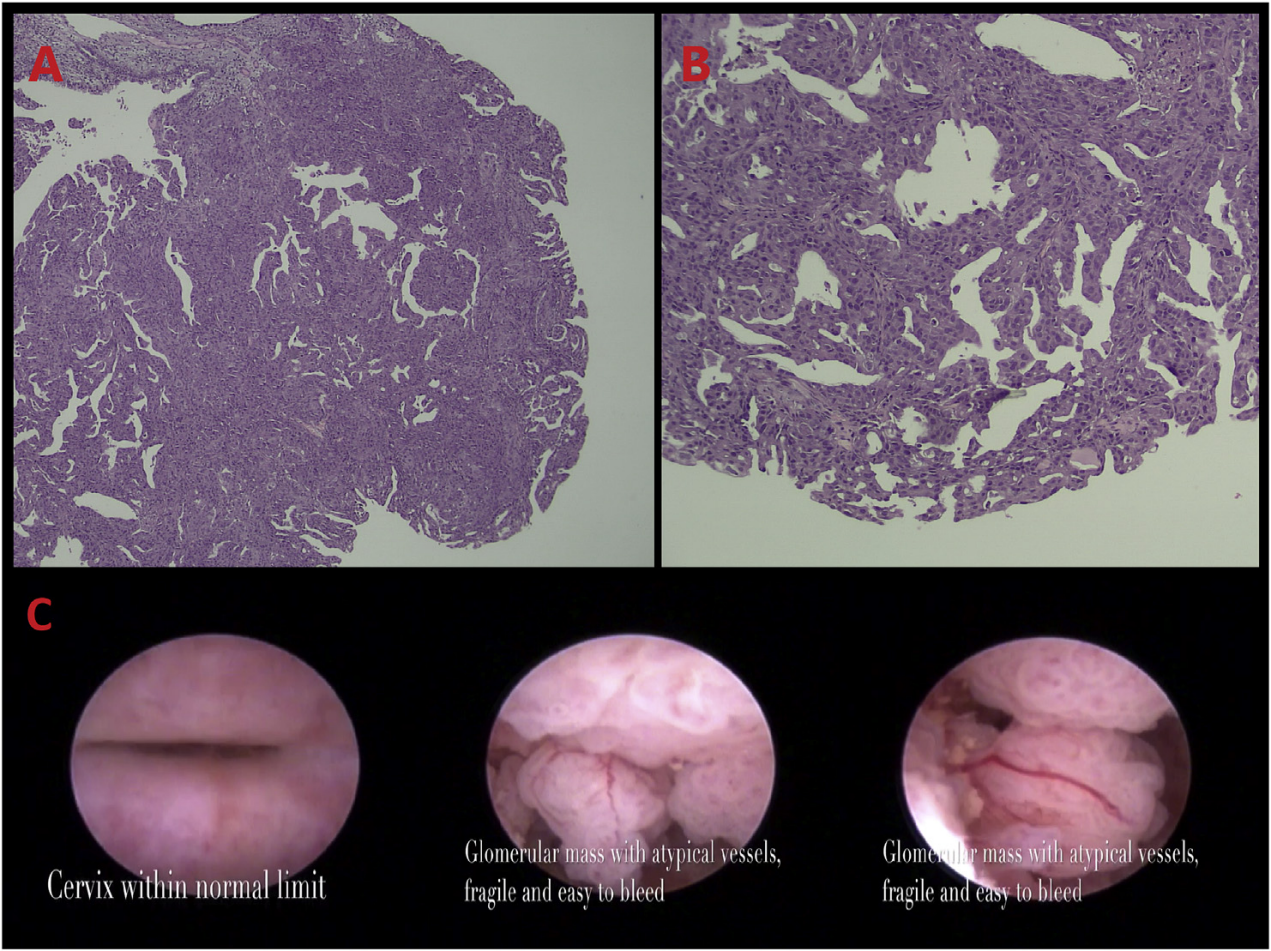
Pre-operative Hysteroscopic findings.

Initial ultrasonography (July 31st, 2018) findings suggested that the uterus cavity was filled with fluid due to blockage at the endocervix. However, the cause could not be determined. Patient was then referred to a tertiary hospital for further investigation. Upon hysteroscopic examination by a gynecology oncology consultant with more than 10 years of experience, several glomerular mass with atypical vessels resembling malignant endometrial lesion were found. The appearance from the biopsy was suggestive for serous Endometrial Carcinoma grade II presumably from the endometrium. There were neither normal endometrial tissue nor hyperplastic zone to be identified (Fig. 1).

Histopathologic specimen showing cancerous cells with no normal tissue (A) 40x zoom and (B) 100x zoom. (C). Glomerular growing mass with atypical vessels.

**Fig. 2:**
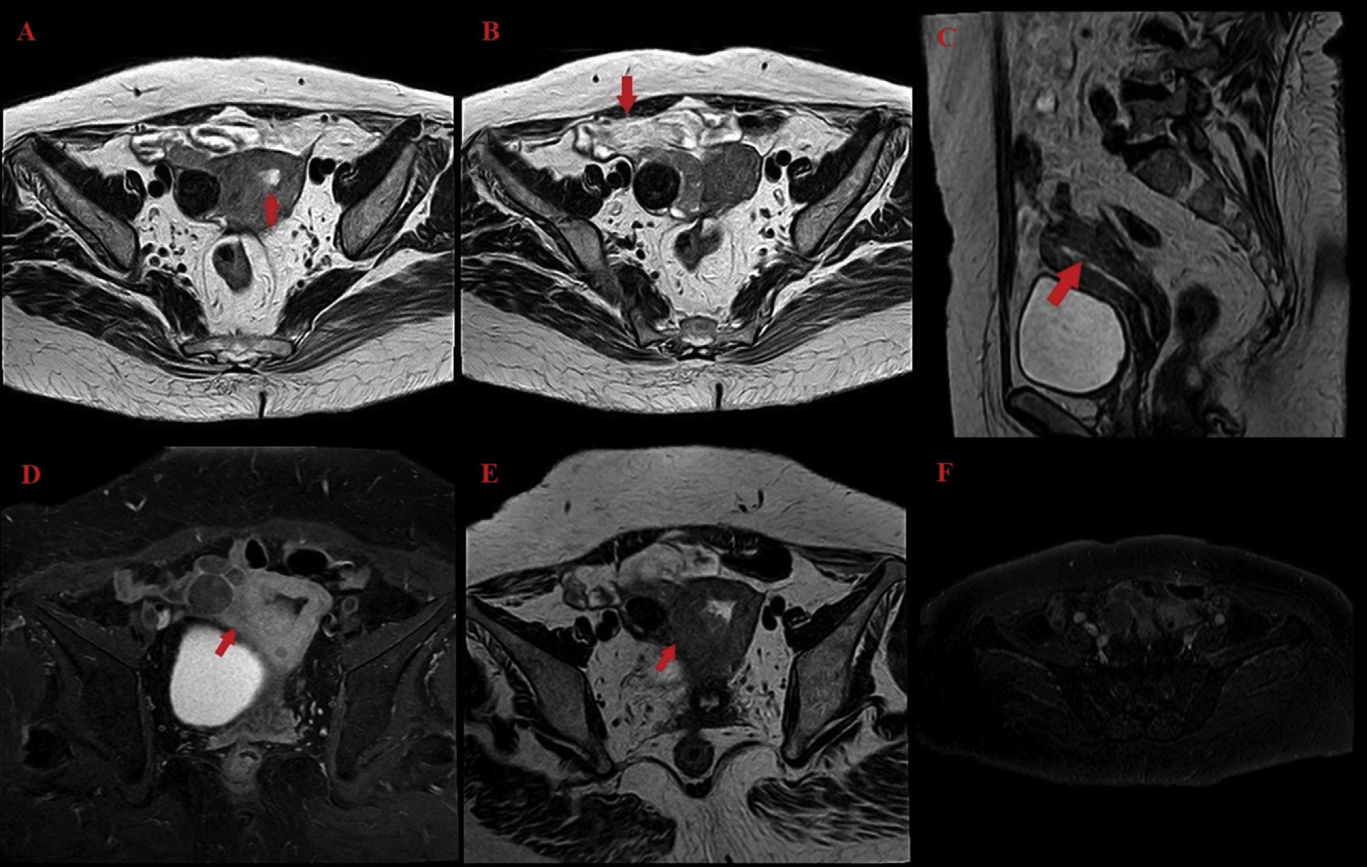
MRI images. (A and B) Axial view showing tumor mass inside the endometrium and at the right fallopian tube. (C) Sagittal view of endometrial tumor. (D and E) T2 and T1 image that showed mild enhancement from contrast administration. (F) Axial view of contrast enhancement.

MRI examination (September 1st, 2018) found two masses: at the tube and endometrium. Endometrial mass was prominent to anterior junctional zone with 13 mm thickness, did not invade the myometrium, and covered <50% of the endometrial surface in accordance to T1A-N0-M0 staging [5]. There were right tubal mass sized 29/30/31 mm and right hydrosalpinx with T2A-N0-M0 staging [6]. There were no signs of metastasis. The right adnexa mass was attached to the right wall of the uterus with suspicion towards ovarian cyst with of 34/37/42 mm, which is not part of the discussion. Other findings were within normal limit (Fig. 2).

Follow-up USG on September 25th, 2018 revealed collapsed uterine cavity. Presence of intra-cavitary malignancy could not be ruled out. There was no widening of parailiac and bilateral of paraaortic lymph nodes.

On October 4th, 2018 surgery was performed by a senior gynecology oncology consultant with more than 15 years of experience. Adhesiolysis and surgical staging laparotomy (total abdominal hysterectomy, bilateral salphingo-oophorectomy, pelvic lymphadenectomy, and para-aorta lymphadenectomy) were conducted.

**Fig. 3:**
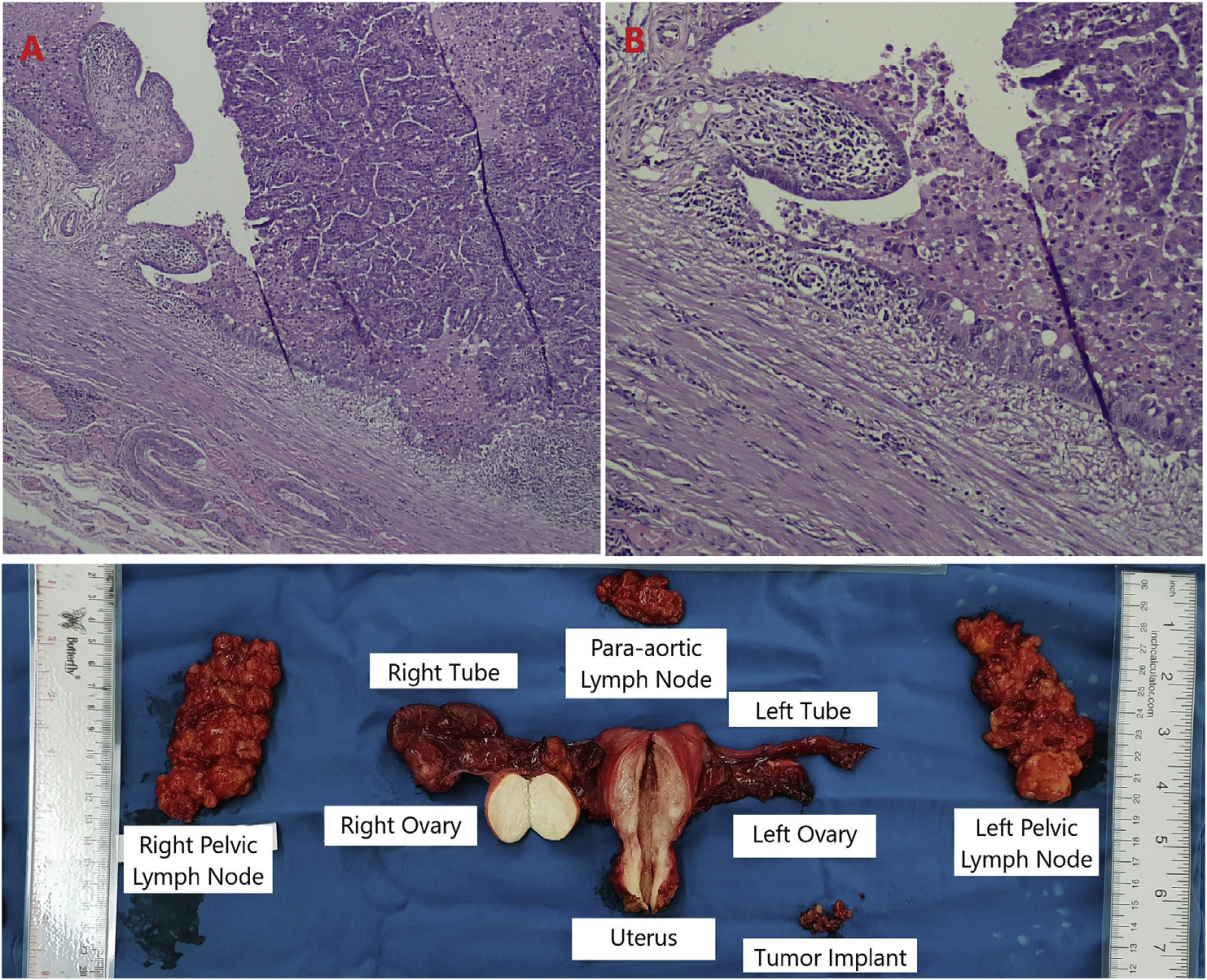
Post-operative Histopathological Examination of Tubal Mass at (A) 40x zoom and (B) 100x zoom showing Carcinoma in Situ. (C) Excised mass.

Macroscopically, there was a normal-sized uterus with thin endometrium and no evidence of tumor in the uterine cavity. The fallopian tumor measured 30/25/20 mm, was brown-yellowish in color but white and fibrous on the inside. From final histopathology expertise, the patient had high-grade serous carcinoma from the right fallopian tube (Fig. 3). Para-aortic, right pelvic, and left pelvic lymph nodes showed histiocytosis sinus and no metastasis. The final diagnosis was then Fallopian Tube Cancer stage IIB.

The patient was then planned to receive three cycles of chemotherapy using carboplatin and paclitaxel. The patient tolerated the chemotherapy well with mild complain of constipation that did not require any further medication to resolve. No recurrence was reported one year after the procedure was done. Regarding the whole experience, the patient understands that FTC is often misdiagnosed due to its rare occurrence and non-distinctive symptoms. The patient is also satisfied that the clinicians have put appropriate efforts to diagnose the origin of the tumor.

## 3 Discussion

Primary adenocarcinoma of the fallopian tube only consists of 0.3%–1.0% of all gynecological cancer [5]. FIGO 2014 has also included FTC classification into a single system together with ovarian and peritoneal cancer. Due to its rare incidence and unspecific symptoms, previous reports have also misdiagnosed the case as other gynecological cancer [7,8].

Hysteroscopy has been reported to have a specificity of 98.1% in diagnosing endometrial carcinoma in post-menopausal bleeding. The false-positive rate should decrease further after biopsy confirmation. The low false positive rate may mislead clinicians to disregard possibilities of spillage from the primary FTC to the otherwise normal endometrial tissue. Use of immunohistochemistry for expression of certain proteins might alleviate this issue [9].

MRI is the modality of choice to diagnose Gynecological Tumors. In this case, enhancement occurs during the administration of contrast which suggests malignant nature. The weakness of the method nevertheless lies in its inability to detect the primary tumor origin. However, several considerations can be taken such as the metastatic stage [10]. Only 30% of patients with stage T1A Endometrial cancer have pelvic lymph node metastasis, while 66% of patients with stage T2 fallopian tube cancer do [11]. Since this patient does not exhibit pelvic lymph node metastasis, endometrial cancer origin is more likely.

To define the tumor origin, the histopathologic examination should be able to visualize the cellular transition from benign to malignant tubal epithelium. The first biopsy from histopathological procedure could not retrieve any normal endometrial tissue. At the age of 74 years old, the patient is expected to have an atrophic endometrial lining. In addition, histologic appearance of type II (serous) carcinoma for both endometrium and fallopian tubes are very similar [12]. Thus, it was very challenging to distinguish the primary cancer origin from the pre-operative measure.

## 4 Conclusion

Despite rigorous examination, the diagnosis of FTC was missed. It was only at the latest stage that the final diagnosis was found. Regardless, a lesson can be learned from this case that due to its unpredictable nature, FTC should not be forgotten as a differential diagnosis. Therefore, the surgeon should always be prepared to adjust the operating procedure in accordance with intra-operative findings.

## Ethical approval

Written informed consent was obtained from the patient for publication of this case report and accompanying images.

No personal detail of the patient has been stated in the manuscript.

## Sources of funding

No external funding received. The study is self-funded.

## Author contribution

Sigit Purbadi, Victor Prana Andika Santawi, and Laila Nuranna designed the case report.

Sigit Purbadi and Victor Prana Andika Santawi wrote the manuscript.

Laila Nuranna, Sahat Matondang, and Hartono Tjahjadi supervised and provided their expertise in this case report.

## Registration of research studies

The manuscript is a case report that does not involve experiments to human participants.

## Consent

Written informed consent was obtained from the patient for publication of this case report and accompanying images.

No personal detail of the patient has been stated in the manuscript.

## Guarantor

Sigit Purbadi.

## Declaration of competing interest

All authors declare that there is no conflict of interest.
